# Plants used in artisanal fisheries on the Western Mediterranean coasts of Italy

**DOI:** 10.1186/1746-4269-9-9

**Published:** 2013-01-28

**Authors:** Valentina Savo, Arianna La Rocca, Giulia Caneva, Fabio Rapallo, Laura Cornara

**Affiliations:** 1 Hakai Network for Coastal People, Ecosystems and Management, Simon Fraser University, 8888 University Drive, Burnaby, BC, V5A 1S6, Canada; 2 Department of Science, University Roma Tre, Viale Marconi 446, I-00146, Rome, Italy; 3 Polo Botanico “Hanbury”, DISTAV, University of Genova, C.so Dogali 1 M, I-16136, Genova, Italy; 4 Department DISIT, University of Piemonte Orientale, Viale Teresa Michel 11, 15121, Alessandria, Italy

**Keywords:** Ethnobotany, Traditional ecological knowledge, Traditional fishery knowledge

## Abstract

**Background:**

Artisanal fisheries in the Mediterranean, especially in Italy, have been poorly investigated. There is a long history of fishing in this region, and it remains an important economic activity in many localities. Our research entails both a comprehensive review of the relevant literature and 58 field interviews with practitioners on plants used in fishing activities along the Western Mediterranean Italian coastal regions. The aims were to record traditional knowledge on plants used in fishery in these regions and to define selection criteria for plant species used in artisanal fisheries, considering ecology and intrinsic properties of plants, and to discuss the pattern of diffusion of shared uses in these areas.

**Methods:**

Information was gathered both from a general review of ethnobotanical literature and from original data. A total of 58 semi-structured interviews were carried out in Liguria, Latium, Campania and Sicily (Italy). Information on plant uses related to fisheries were collected and analyzed through a chi-square residual analysis and the correspondence analysis in relation to habitat, life form and chorology.

**Results:**

A total of 60 plants were discussed as being utilized in the fisheries of the Western Italian Mediterranean coastal regions, with 141 different uses mentioned. Of these 141 different uses, 32 are shared among different localities. A multivariate statistical analysis was performed on the entire dataset, resulting in details about specific selection criteria for the different usage categories (plants have different uses that can be classified into 11 main categories). In some uses, species are selected for their features (e.g., woody), or habitat (e.g., riverine), etc. The majority of uses were found to be obsolete (42%) and interviews show that traditional fishery knowledge is in decline. There are several reasons for this, such as climatic change, costs, reduction of fish stocks, etc.

**Conclusions:**

Our research correlates functional characteristics of the plants used in artisanal fishery and habitats, and discusses the distribution of these uses. This research is the first comprehensive outline of plant role in artisanal fisheries and traditional fishery knowledge in the Mediterranean, specifically in Italy.

## Background

Artisanal fisheries can be found all over the world and with great variety and regionalism to their utilization. "Traditional Fishery Knowledge" refers to the practices of fishermen and fisheries that have evolved over the millennia, it relies on the use of natural materials for construction of tools, vessels and equipment, and observations of weather patterns, sea conditions, etc., and the accumulation and transmission of that knowledge as it relates to fishing and fishing related activities. In the Mediterranean region it differs from one country to another and encompasses diverse methods. Moreover, it follows different and unpredictable trends (economy, local regulations, etc.): thus, it can be difficult to assess its status over time [1]. However, the activities and practices associated with artisanal fisheries are declining within the Mediterranean basin, mainly due to overfishing [2], which has led to a reduction of fish stocks [3,4]. Other contributing factors include an increase in costs (fuel, equipment) and a reduction of available manpower [5], due to lack of interest in these occupations among younger generations.

Modern commercial fishing in the Mediterranean basin is carried out by well-equipped fishing vessels, new technologies are used to locate fish stocks, pull fishing nets and to preserve fish on board. At the same time, newer materials have replaced plant fibers and wood used in making boats and fishing equipment, as they are cheaper to produce. Thus, traditional instruments, tools, ships, fishing strategies and their related knowledge are rapidly disappearing. This cultural erosion of traditional knowledge relating to fishery is also happening in other parts of the world (e.g., [6,7]). However, the ethnobotanical knowledge related to traditional fishery has been poorly investigated the world over (e.g., [6,8-10]). These studies are generally focused on specific practices or topics (e.g., canoe construction, mangrove exploitation, natural resource management). This research is the first comprehensive outline of plants role in artisanal fisheries and traditional fishery knowledge in the Mediterranean, specifically in Italy.

We hypothesized that plants used in fishery would be chosen for specific features (e.g., flexibility, robustness, etc.), but also for reasons related to cultural preference. Indeed, there are many reasons why particular plant species may be chosen for a specific use and several theories have been hypothesized to account for these choices (e.g., the apparency theory, [11-13]). Selection criteria for plants used in fishing practices have never been analyzed. There have been some suggestions however, such as those regarding canoe construction put forth by [14] and [15].

The aims of our research are to:

● Record and review present knowledge on ethnobotanical uses of plants in traditional fishery in the Western Mediterranean coastal regions of Italy.

● Analyze possible patterns of diffusion of ethnobotanical uses of plants in fishery in this area.

● Define to what extent the intrinsic properties of plants and their ecology can affect the selection process.

As both ecological and cultural factors may affect the traditional use of a plant in a dynamic process, we analyzed the diffusion of uses, habitat, chorology and life forms of the plants used in artisanal fishery to determine if there is an ecological-functional *vs* cultural pattern to their selection. Artisanal fisheries are in decline, as such we expect to find that uses of plants for this activity are 1) also in decline, 2) poorly documented in the literature, and 3) evenly distributed across regions of the coasts.

## Methods

This ethnobotanical study includes data from bibliographic sources and original field data, which were merged together (Additional file 1) for qualitative analyses.

All available literature sources (books, research papers, local publications, reviews) dealing with ethnobotany in the Western coastal regions of Italy were screened for uses of plants related to fishery activities. Specifically, our focus was on mention of plants used in the construction of ships, fishnets, fish traps, ropes, etc.

**Figure 1:**
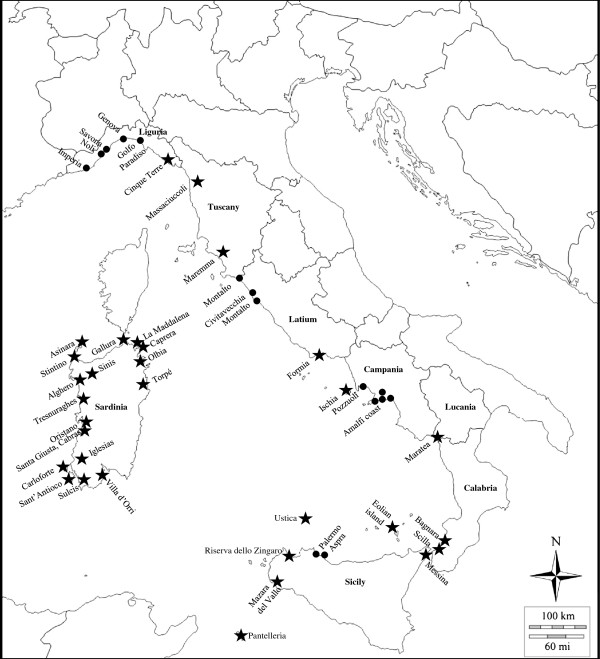
**Geographic position of localities with available data.** A star (★) indicates bibliographic sources and a point (●) the localities of interviews.

Original data were obtained through interviews with fishermen, sailors and boat builders, and also with residents of coastal and semi-coastal villages. The field research was carried out between 2007 and 2011 in different localities in the prefectures of Genoa, Imperia, Savona, Viterbo, Rome, Naples, Salerno and Palermo (Figure 1).

During the field work, 58 semi-structured interviews were performed with people born or having spent most of their lives in the research areas. Informants were entirely male with their ages ranging from 30 to 89 (average age of about 64 years). Most informants were people who worked in fishery or other maritime activities, although some interviews were carried out with people who had other occupations. Interviews were mainly carried out in harbors, sailors’ meeting places, fish markets and traditional shipyards. The number of interviews and their duration were entirely dependent on the availability of informants.

Before starting an interview, the scope of this study was explained to the possible informant and Prior Informed Consent [16] was verbally requested. Semi-structured interviews were conducted following the ISE Code of Ethics [17]. Informants were first asked a few demographic questions (age, job, place of residence) followed by questions about plants used in fishery. These semi-structured interviews consisted of the following questions:

Have you ever used plants in fishing related activities? Which plants? How and how much have you used this plant? Which part of the plant have you used? Have you used this plant in association with another plant? Do you still use this plant? General comments on the status of fishery were also noted when provided by informants.

Plants mentioned and shown by informants were collected and vouchers are preserved at the Herbarium of the Science Department of the University Roma Tre (URT) and at the Herbarium of the University of Genova (GE) [18]. Plant species were identified using the “Flora d’Italia” [19] and their scientific names were updated [20,21]. Plant taxonomy at higher levels follows APG III [22].

Informants often presented materials and products (e.g., ropes, pieces of wood) but they were also asked to indicate the plant in the wild. However, in some cases it was not possible to collect the complete voucher of the related plant material. In these cases it was only possible to identify the plants to the level of genus.

Both uses reported in our literature review and those obtained through field work were merged together in order to define an overview of the diversity of uses and their distribution patterns in the Western Mediterranean coastal regions of Italy.

The loss of ethnobotanical knowledge related to artisanal fishery was evaluated considering the currency of use. However, this type of analysis was only possible for data obtained through field research. All plant species were categorized by their usage, aggregated into 11 main categories [BAR (Barrels); BAS (Basketry); AQC (Aquaculture); DYE (Dyeing); FNT (Fish nets); FTR (Fish traps); FSH (Fishing); ILF (Illegal fishing); SHR (Shrouds); SHB (Ship building); TOL (Tools)]. Plants were then characterized from a structural (life form) and ecological (chorology, habitat of gathering) perspective (according to [19]). Afterwards, the geographic distribution of each use, life form, chorology and habitat of species for each usage category were statistically analyzed in order to highlight possible patterns of plant selection. This multivariate analysis was performed on explanatory variables [taxonomic group, habitat, region, life form and chorology) vs. usage categories (see Table 1)]. The data set consisted of five two-way contingency tables that were considered separately. In these five tables, the plant species counts (within each main category) versus the explanatory variables were displayed. Data were analyzed using two different methods:

(a) The analysis of the standardized chi-square residuals under independence. In particular, a large residual for a cell implies that the observed count is much larger than expected, meaning that the given cell represents a remarkable combination.

(b) The correspondence analysis, when the quality of the representation over the first two axes was satisfactory [23]. Using the software R [24] with the package “ca”, we drew the projection of the row and column levels onto the best coordinate plane. We used the asymmetric row plot in order to keep the “Usage category” variable as the response variable. As correspondence analysis is based on a projection of the rows and columns from a multidimensional space onto a plane, not all projected points offer a good representation of the data. Data-points with low quality were not considered for comments. For well-represented points, the interpretation of such plots is as follows: a) proximity of row points means similarity of the corresponding row profiles, while b) proximity of a column point to a row point must be read as a major interaction between this row and column.

**Table 1:** Main usage categories of plant species and regions where the use is shared

**Use (number of species or taxa)**	**Usage category (abbrev.)**	**Species**	**Region**
Bait (3)	FSH	*Brassica oleracea* L.	Liguria
		*Helichrysum italicum* (Roth) G. Don	Liguria, Sardinia
		*Myrtus communis* L.	Campania
Barrels (6)	BAR	*Castanea sativa* Miller	Campania, Liguria, Sicily
		*Fagus sylvatica* L.	Liguria
		*Fraxinus ornus* L.	Sicily
		*Quercus pubescens* Willd. s.l.	Sicily
		*Quercus robur* L. s.l.	Sicily
		*Salix* sp.	Sicily
Basketry (13)	BAS	*Ampelodesmos mauritanicus* (Poiret) T. Durand et Schinz	Latium
		*Arundo donax* L.	Campania
		*Arundo plinii* Turra	Campania
		*Castanea sativa* Miller	Campania, Liguria
		*Erica arborea* L.	Campania
		*Juncus* sp. pl.	Campania, Sardinia
		*Myrtus communis* L.	Sardinia
		*Olea europaea* L.	Sardinia
		*Pistacia lentiscus* L.	Sardinia
		*Quercus suber* L.	Liguria, Sardinia
		*Salix alba* L.	Liguria
		*Schoenoplectus lacustris* (L.) Palla	Sardinia
		*Tamarix gallica* L.	Campania
Caulking (2)	SHR	*Cannabis sativa* L.	Liguria
		*Gossypium* sp. pl.	Liguria
Coloring (1)	DYE	*Linum usitatissimum* L.	Campania, Sicily
Dams of fish ponds (2)	AQC	*Arundo donax* L.	Sardinia
		*Phragmites australis* (Cav.) Trin.	Sardinia
Dyeing of fish nets (7)	DYE	*Alnus glutinosa* (L.) Gaertn.	Tuscany
		*Castanea sativa* Miller	Liguria
		*Pinus* sp.	Liguria
		*Pinus* sp. pl.	Campania, Latium, Sicily
		*Pinus halepensis* Mill.	Campania, Sicily
		*Pinus pinea* L.	Liguria
		*Pistacia lentiscus* L.	Sicily
Fish nets (9)	FNT	*Ampelodesmos mauritanicus* (Poiret) T. Durand et Schinz	Liguria
		*Arundo donax* L.	Campania
		*Cannabis sativa* L.	Liguria, Latium, Sardinia, Sicily
		*Castanea sativa* Miller	Liguria
		*Chamaerops humilis* L.	Sardinia
		*Cocos nucifera* L.	Liguria
		*Gossypium* sp. pl.	Campania, Latium, Liguria, Sardinia, Sicily
		*Lygeum spartum* L.	Sardinia
		*Phormium tenax* J.R. et G. Forster	Sardinia
Fish traps (19)	FTR	*Ampelodesmos mauritanicus* (Poiret) T. Durand et Schinz	Latium
		*Arundo donax* L.	Campania, Sardinia
		*Arundo plinii* Turra	Campania
		*Castanea sativa* Miller	Liguria
		*Erica arborea* L.	Campania
		*Fraxinus ornus* L.	Liguria
		*Gossypium* sp. pl.	Latium, Liguria, Sardinia
		*Juncus* sp.	Campania, Latium, Liguria, Sicily
		*Juncus* sp. pl.	Campania, Sardinia
		*Juncus acutus* L.	Sardinia
		*Juncus maritimus* Lam.	Sardinia
		*Myrtus communis* L.	Latium, Sardinia
		*Myrtus communis* L. subsp. *communis*	Campania
		*Olea europaea* L.	Campania
		*Pistacia lentiscus* L.	Campania
		*Punica granatum* L.	Sardinia
		*Salix* sp.	Liguria
		*Salix alba* L.	Campania
		*Tamarix gallica* L.	Campania
Fishing (6)	FSH	*Gossypium* sp. pl.	Campania, Liguria
		*Laurus nobilis* L.	Campania
		*Olea europaea* L.	Sardinia, Sicily
		*Chamaerops humilis* L.	Sicily
		*Euphorbia characias* L.	Liguria
		*Juniperus* sp. pl.	Sardinia
Floats (1)	FSH	*Quercus suber* L.	Campania, Latium, Liguria, Sardinia, Sicily
Grill (1)	TOL	*Castanea sativa* Miller	Liguria
Hooks (1)	FSH	*Acacia karroo* Hayne	Sardinia
Illegal fishing (5)	ILF	*Daphne gnidium* L.	Tuscany
		*Euphorbia dendroides* L.	Sicily
		*Euphorbia helioscopia* L.	Sicily
		*Pistacia lentiscus* L.	Tuscany
		*Verbascum thapsus* L.	Tuscany
Making ships go faster (1)	SHB	*Opuntia ficus-indica* (L.) Miller	Campania
Mussel farming (3)	AQC	*Ampelodesmos mauritanicus* (Poiret) T. Durand et Schinz	Liguria
		*Castanea sativa* Miller	Campania
		*Quercus ilex* L.	Sardinia
Pulleys (1)	TOL	*Pyrus communis* L.	Liguria
Ramps (6)	TOL	*Citrus aurantium* L.	Campania
		*Citrus limon* (L.) Burm. f.	Campania
		*Cormus domestica* (L.) Spach	Campania
		*Olea europaea* L.	Campania
		*Quercus ilex* L.	Campania, Sicily
		*Quercus pubescens* Willd. subsp. *pubescens*	Campania
Ropes (8)	SHR	*Agave americana* L.	Sicily
		*Ampelodesmos mauritanicus* (Poiret) T. Durand et Schinz	Latium, Liguria, Lucania, Sicily
		*Cannabis sativa* L.	Sardinia, Sicily
		*Chamaerops humilis* L.	Sardinia
		*Cocos nucifera* L.	Liguria, Sardinia, Sicily
		*Juncus* sp. pl.	Sardinia
		*Lygeum spartum* L.	Sardinia
		*Phormium tenax* J.R. et G. Forster	Sardinia
Ship building (33)	SHB	*Agave americana* L.	Sicily
		*Alnus glutinosa* (L.) Gaertn.	Liguria
		*Arbutus unedo* L.	Liguria
		*Arundo donax* L.	Sardinia
		*Castanea sativa* Miller	Campania, Liguria
		*Ceratonia siliqua* L.	Campania
		*Cormus domestica* (L.) Spach	Campania
		*Fagus sylvatica* L.	Campania, Liguria
		*Fraxinus ornus* L.	Liguria
		*Fraxinus ornus* L. subsp. *ornus*	Campania
		*Juglans regia* L.	Liguria
		*Juncus acutus* L.	Sardinia
		*Juniperus* sp. pl.	Sardinia
		*Laburnum anagyroides* Medik	Liguria
		*Larix decidua* Mill.	Liguria
		*Mespilus germanica* L.	Sicily
		*Morus* sp.	Calabria, Liguria
		*Morus alba* L.	Campania, Sicily
		*Olea europaea* L.	Liguria
		*Picea abies* (L.) H. Karst.	Liguria
		*Pinus* sp.	Calabria, Liguria
		*Pinus halepensis* Mill.	Sardinia, Sicily
		*Pinus nigra* J.F. Arnold s.l.	Sardinia
		*Pinus pinaster* Aiton s.l.	Liguria
		*Pinus pinea* L.	Campania, Liguria
		*Prunus avium* (L.) L.	Liguria
		*Quercus* sp.	Calabria, Liguria
		*Quercus ilex* L.	Campania, Liguria
		*Quercus pubescens* Willd. subsp. *pubescens*	Campania
		*Quercus robur* L. s.l.	Liguria
		*Robinia pseudoacacia* L.	Liguria
		*Schoenoplectus lacustris* (L.) Palla	Sardinia
		*Ulmus minor* Miller s.l.	Campania, Liguria
Ship models (1)	SHB	*Picea abies* (L.) H. Karst.	Liguria
Shrouds (1)	SHR	*Chamaerops humilis* L.	Sardinia
Tools (10)	TOL	*Arbutus unedo* L.	Liguria
		*Arundo donax* L.	Campania
		*Castanea sativa* Miller	Liguria
		*Fagus sylvatica* L.	Liguria
		*Fraxinus ornus* L.	Liguria
		*Myrtus communis* L.	Sardinia
		*Olea europaea* L.	Latium
		*Phillyrea angustifolia* L.	Sardinia
		*Tamarix africana* Poir.	Sardinia
		*Tamarix gallica* L.	Sardinia
Waterproofing (1)	DYE	*Linum usitatissimum* L.	Liguria

**Notes: BAR:** Barrels (different parts of the barrels used for preserving fish); **BAS:** Basketry (baskets, trays and cages for fish); **AQC:** Aquaculture (dams for fish ponds and mussel farming); **DYE:** Dyeing (dyeing of fish nets, waterproofing and painting of boat parts); **FNT:** Fish nets (various parts of different kinds of fish nets); **FTR:** Fish traps (various parts of fish traps); **FSH:** Fishing (hooks, baits, floats, etc.); **ILF:** Illegal fishing (poisonous plants used to narcotize or catch fish); **SHR:** Shrouds (various ropes and shrouds); **SHB:** Ship building (different parts of boats: e.g., hull, keel, upperworks, transverse frames, etc.); **TOL**: Tools (different tools used in fishing: e.g., needles for repairing fish nets, grill for hanging fish nets, ramps and pulleys for pulling boats out of the water).

## Results

In the investigated areas, as well as in other Italian locations, uses of plants involved in fishery are poorly investigated, as shown by the relatively few data that were obtained from bibliographic sources (considering reports from Liguria, Tuscany, Latium, Campania, Lucania, Calabria, Sardinia and Sicily).

Informants (considering only our original field data) were mainly elders (over 60 years) (62%), with a lower percentage of informants being middle aged (40–60 years) (29%), while a small percentage (9%) of informants were under 40 years old. Most informants were fishermen (47%); other informants were ship builders (9%), pensioners (8%) (mainly retired fishermen), dockworkers (7%), fish sellers (3%), and sailors (2%). The remainder (24%) was involved in non-maritime occupations.

A total of 60 species of plants (*plus* three subspecies, 7 taxa at the genus level, with a total of 32 families) (Additional file 1) were found to be used in fishery activities. However, very few data on artisanal fishery were found for the Calabria region, even though fishing and its related crafts are an important commercial activity in the region [25]. A total of 265 different mentions of uses were recorded, of which 162 were from interviews. The cited taxa can be grouped into 11 usage categories, although specific differences can exist among uses (141 different plant uses, Table 1). A total of 32 uses are shared among different localities (within or outside the same region), while the remaining ones are more localized.

**Figure 2:**
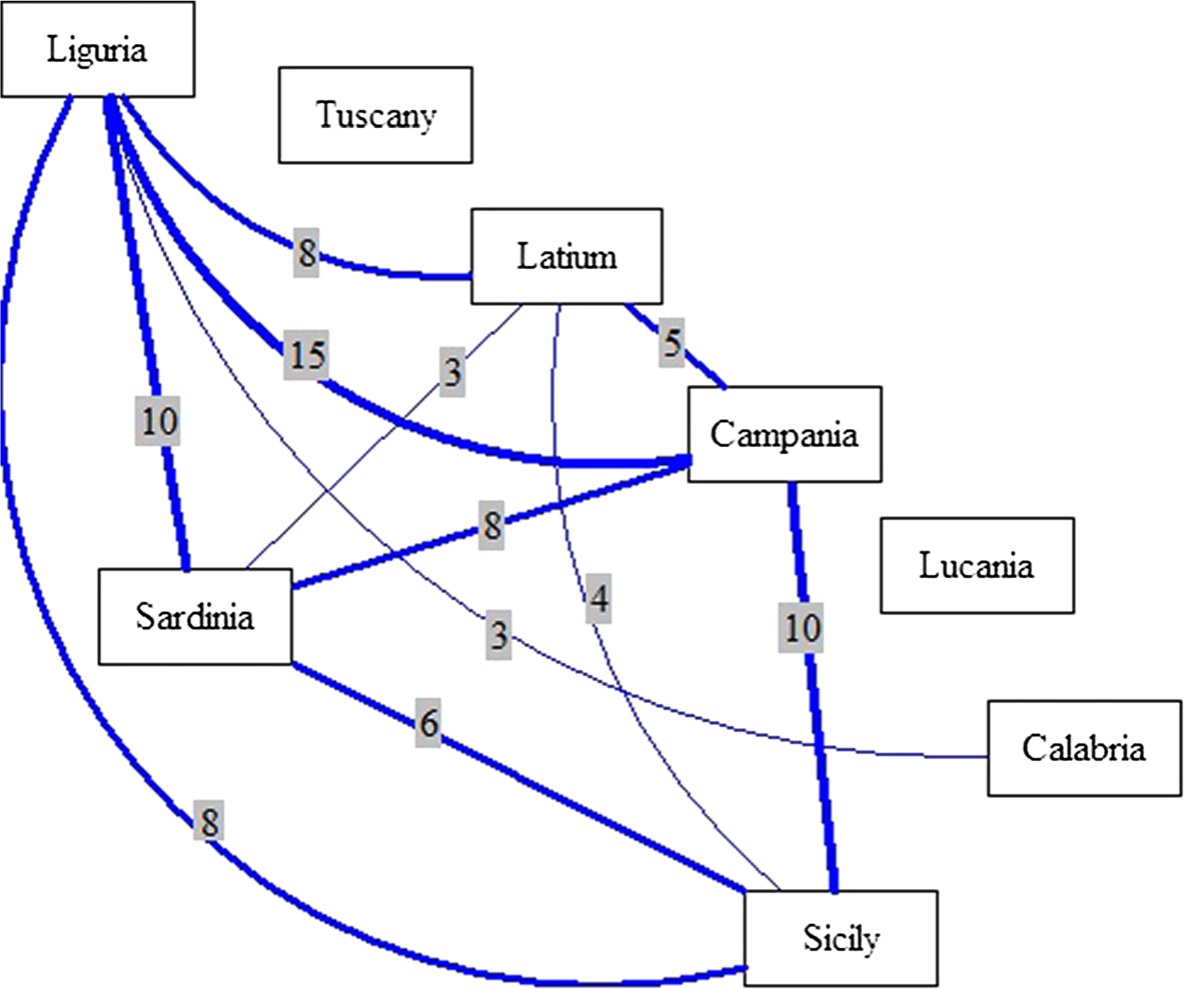
Main connections among regions considering the number of shared uses.

The use of *Ampelodesmos mauritanicus* (Poiret) T. Durand et Schinz for weaving ropes, for example, is shared among various localities in Liguria, Latium, Campania, Lucania and Sicily; the use of *Quercus suber* L. for making floats was reported in various localities in Liguria, Latium, Campania, Sardinia and Sicily. In Figure 2 the major connections (more than five shared uses) among Western Italian coastal regions are shown.

Some uses are more localized and specific: for example the use of cladodes of *Opuntia ficus-indica* (L.) Miller which are rubbed on the hull of ships in order to make them go faster in the Amalfi Coast (Campania), *Brassica oleracea* leaves are used as a bait in Camogli (Liguria), the wood of *Olea europaea* is carved for making sewing implements in Marina di Montalto (Latium).

The most ethnobotanically represented families are Fagaceae, Pinaceae and Poaceae, while the majority of families (17) are represented by a single species.

The analysis of the life form spectrum shows that the complete set of species consists primarily of phanaerophytes (77%) and secondly by hemicryptophytes (8%), geophytes (7%), therophytes (5%) and chamaephytes (3%).

The analysis of the chorotype of the complete set of species highlights a prevalence of Mediterranean plants (45%) [(35% Steno-Mediterranean, (10%) Euro-Mediterranean]. Some plants have a European (18%) or a wide distribution (8%), while endemic or sub-endemic species are lacking. Some plants are naturalized (6%) or cultivated (17%).

Plants have the following habitat distribution (the same species may be found in one or more habitat): maquis and garigues (25 species), Mediterranean woodlands (14 species), home gardens and cultivated land (12 species), river banks (9 species), arid meadows and sunny rocky places (9 species), temperate woodlands (7 species), maritime habitats (dunes, halophylous rocks) (6 species) and uncultivated land (5 species).

Phanerophytes are generally used as timber for ship building (25 species), for making tools (10), fish traps (10), ramps (6) and barrels (cooperage) (5); hemicryptophytes are used for fish nets and/or fish traps (2); geophytes are mainly used for fish traps (3) or dams (2) and chamaephytes are used for preparing baits (2). Many species occurring on river banks are used for fish traps (5) or basketry (4 species); while many species of the Mediterranean maquis and garigues are used to weave fish traps (6). The majority of plants growing in the Mediterranean woodlands are used for ship building (10), while all the species of the temperate woodlands (7) are used for this purpose. Plants growing in other habitats are used for various purposes.

The results of the bivariate analysis are summarized in Table 2, where the standardized chi-square residuals are reported. The standardized residuals greater than two are in bold. When appropriate, the plot of the correspondence analysis is also considered.

**Table 2:** The bivariate analysis (with the standardized chi-square residuals) of uses in relation to selection/distribution variables

		**Usage category**										
		**BAR**	**BAS**	**AQC**	**DYE**	**FNT**	**FTR**	**FSH**	**ILF**	**SHR**	**SHB**	**TOL**
**Habitat**	Temperate woodlands	1.33	−0.56	0.37	0.94	−0.04	−0.86	−1.22	−0.90	−0.96	1.65	−0.40
	Cultivated land	−0.79	−1.16	−0.73	1.33	**2.44**	−1.34	−0.15	−0.79	**2.69**	−0.80	0.65
	Maquis and garigues	−0.74	0.75	0.17	−0.40	−0.40	0.04	1.11	−0.04	−0.88	−0.44	0.47
	Mediterranean woodlands	1.43	−0.52	−0.14	−0.56	−0.56	−0.94	0.95	−1.16	−0.43	0.91	0.27
	Along rivers	0.21	1.72	0.37	−0.04	−1.02	**2.43**	−1.22	−0.90	−0.96	−0.71	−0.40
	Maritime habitats (dunes, halophylous rocks)	−0.67	0.04	−0.62	0.56	−0.76	1.52	−0.91	0.82	−0.72	−0.32	0.43
	Uncultivated land	−0.56	−0.82	−0.52	−0.64	−0.64	0.11	−0.76	**3.01**	1.07	0.94	−1.02
	Arid meadows and sunny rocky places	−0.76	−0.22	0.71	−0.87	1.44	−0.52	0.88	1.85	1.63	−1.25	−0.66
**Region**	Liguria	−0.18	−0.83	−0.54	0.53	0.08	−1.05	0.08	−1.19	−0.73	**2.09**	−0.17
	Sardinia	−1.37	1.02	**2.20**	−1.49	0.52	0.57	0.52	−1.08	1.17	−1.20	−0.21
	Sicily	**4.08**	−1.44	−0.85	0.65	−0.05	−1.31	−0.05	1.78	1.47	−0.72	−0.86
	Campania	−0.72	1.29	−0.42	0.33	−0.63	1.09	−0.15	−1.13	−1.52	−0.56	1.45
	Latium	−0.73	−0.12	−0.63	−0.12	0.83	1.58	−0.12	−0.57	−0.05	−0.63	−0.23
	Lucania and Calabria	−0.40	−0.59	−0.35	−0.59	−0.59	−0.75	−0.59	−0.32	1.18	**2.09**	−0.62
	Tuscany	−0.40	−0.59	−0.35	1.11	−0.59	−0.75	−0.59	**9.10**	−0.57	−0.98	−0.62
**Taxonomic Group**	Gymnosperms	−0.77	−1.14	−0.70	**2.21**	−0.95	−1.37	0.01	−0.70	−0.89	**3.06**	−1.34
	Early div. Angiosperms	−0.21	−0.30	−0.19	−0.32	−0.25	−0.37	**3.49**	−0.19	−0.24	−0.49	−0.36
	Rosids	1.20	−0.54	−0.29	1.20	−0.67	−0.43	0.05	0.99	−1.47	0.09	0.06
	Asterids	0.02	0.60	−0.90	−1.51	−1.21	0.51	0.29	0.20	−1.14	−0.66	**2.96**
	Monocots	−1.20	1.05	1.63	−1.84	**2.60**	1.13	−0.91	−1.10	**3.65**	−1.47	−1.60
**Life form**	P	0.69	−0.57	−0.91	0.08	−0.56	−0.88	0.17	−0.40	−0.83	1.06	0.93
	H	−0.82	1.28	0.59	−1.00	1.37	0.60	−1.06	0.59	**2.22**	−0.93	−1.42
	G	−0.70	0.90	**2.48**	−0.86	0.56	1.95	−0.91	−0.64	−0.81	−1.07	−0.39
	CH	−0.30	−0.44	−0.27	−0.37	−0.32	−0.53	**4.79**	−0.27	−0.35	−0.71	−0.52
	T	−0.47	−0.70	−0.43	**2.87**	−0.51	0.35	−0.61	1.88	1.28	−1.13	−0.82
**Chorology**	Mediterranean	−1.56	0.89	−0.28	−0.22	−0.22	0.83	1.73	0.36	−0.22	−1.21	0.08
	European	**3.24**	−0.64	−0.27	0.13	−1.35	−0.86	−1.53	−0.27	−1.35	**2.21**	−0.32
	Wide	−0.68	0.85	**2.26**	−0.80	0.44	0.62	−0.91	0.79	−0.80	−0.46	−0.51
	Naturalized	−0.46	−0.71	−0.46	−0.54	−0.54	0.54	−0.61	−0.46	1.30	0.66	0.28
	Cultivated	−0.77	−1.19	−0.77	1.30	**2.40**	−1.28	−0.06	−0.77	**2.40**	−0.81	0.61

**Notes: BAR**: Barrels; **BAS**: Basketry; **AQC**: Aquaculture; **DYE**: Dyeing; **FNT**: Fish nets; **FTR**: Fish traps; **FSH**: Fishing; **ILF**: Illegal fishing; **SHR**: Shrouds; **SHB**: Ship building; **TOL**: Tools.

In bold, the values higher than 2.0.

The following summaries are from the results of the bivariate analysis. Words appearing in quotations correspond with their references in Table 2. These findings show that:

● Usage category *vs* Habitat: cultivated areas are the preferred habitats of plants involved in the construction and implementation of “Fish nets” and “Shrouds”, river banks (or “Along river”) are the habitat of plants relating to “Fish traps”, while the “Uncultivated” habitat is more important for plants used in activities described as “Illegal fishing”. Many plants growing along rivers are known to have flexible stems. This is probably an adaptation to the mechanical action of flowing water. Other plant species relating to traditional fishery are mostly cultivated. In this relation, the correspondence analysis did not yield satisfactory results.

**Figure 3:**
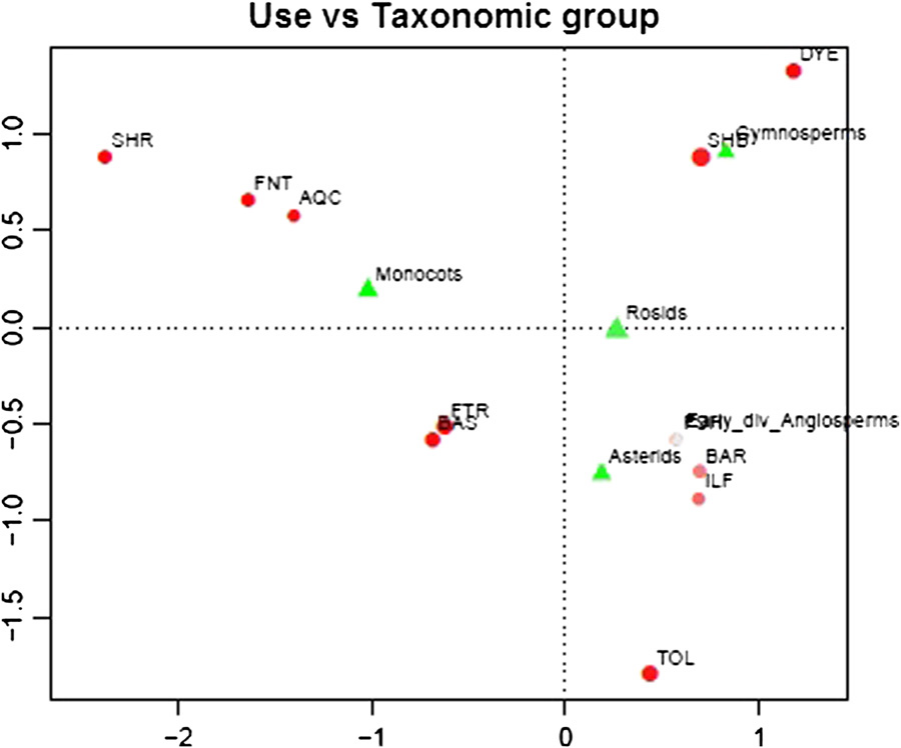
**Correspondence analysis for analyzing the relation Usage category/Phylogeny.** The mass of each level is proportional to the diameter of the point, while its quality is proportional to the color intensity.

● Usage category *vs* Taxonomic group: the results of this analysis show that gymnosperms are especially linked with “Dyeing of fish nets” and “Ship building”, Early div. angiosperms with “Fishing”, while asterids are generally selected for “Tools” and monocots for “Fish nets” and “Shrouds”. Gymnosperms are generally used for building of ship hulls since they are resinous and resistant to salt water. In other parts of the world (e.g., Fiji), species for hull building are selected for their hard and durable wood [14]. The bark of gymnosperms is also rich in tannins and commonly used for strengthening and coloring nets and other fibers to be used in the water. In India, the bark of a completely different species *Ceriops decandra* (Griff.) W. Theob.] is used for the same purpose [9] since it is rich in tannins [26]. Monocots include several species that are flexible but resistant (such as many Poaceae species) and are used for nets and shrouds. The Correspondence Analysis gives a good representation of data, and the plot is displayed in Figure 3 (the inertia on the two axes is 82%). This plot shows one more connection, namely between monocots and “Aquaculture”.

● Usage category *vs* Life form: the residuals show that hemicryptophytes are mainly chosen for making “Shrouds”, geophytes are prevailing in “Aquaculture” and therophytes in “Dyeing of fish nets”. Moreover, there is a remarkable connection between chamaephytes and “Fishing”. In this case, the analysis highlights species that are closely linked to a specific usage category: for example, the majority of species (31 out of 34) for shipbuilding are woody, but woody species are also selected for tools or in other categories. The Correspondence Analysis for this table did not generate significant results.

**Figure 4:**
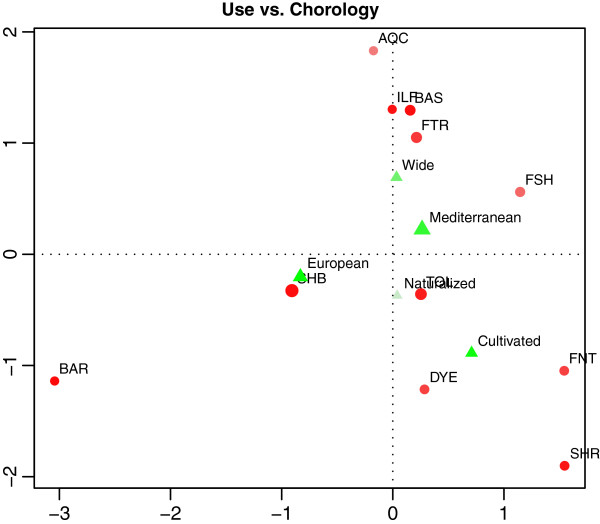
**Correspondence analysis for analyzing the relation Usage category/Chorology.** The mass of each level is proportional to the diameter of the point, while its quality is proportional to the color intensity.

● Usage category *vs* Chorology: plants for making “Barrels” and used in “Ship building” have mainly an “European” distribution, while plants used in aquaculture have a “Wide” distribution. Moreover, plants for “Fish nets” and “Shrouds” are “Cultivated”, thus they can have various origins. In this case, it is noteworthy that “European” species are mostly trees and are used for making barrels and ship building. Interestingly, despite these plants having a “European” distribution, barrels relating to traditional fishery are mainly produced in Sicily. The plot of the Correspondence Analysis is presented in Figure 4 (the inertia on the two axes is 82%), and it supports the findings discussed above.

The analysis of the Usage category *vs* Region is reported in Table 2. However, studies reported in bibliography have an uneven distribution, making the statistical interpretation of this analysis less sound. Indeed, certain usage categories were reported only in a specific area (or region), or in certain regions we only have data on a specific usage category: for example in Calabria we only had data on shipbuilding.

**Figure 5:**
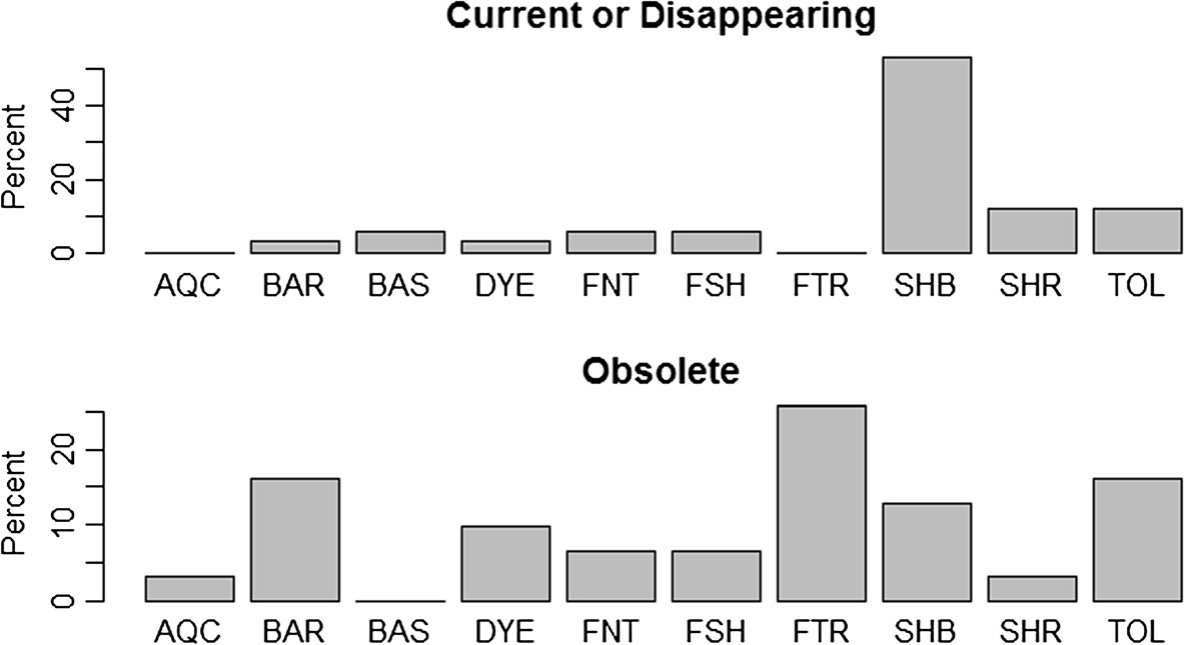
**Barplots of the usage categories.** Current or disappearing uses (top) and Obsolete uses (bottom).

Finally, in order to assess the currency of the recorded uses we excluded bibliographic sources, since from these sources it was not always possible to define if a plant was still in use or not. Findings from our field work indicate that the majority of artisanal fishery uses of plants are no longer practiced (42% of uses are obsolete). Nevertheless, some uses are still practiced (28%), while others are disappearing (7%), (the remaining 23% of cases had missing data). Based on the data from interviews, we show the evolution of the uses through a diagram. We built two barplots of the usage categories for obsolete uses and current or disappearing uses, separately. The plots are displayed in Figure 5. While for obsolete uses there is a wide spectrum of usage categories, nowadays the uses still practiced refer mainly to three usage categories: Ship building, Tools, and Basketry.

## Discussion

In Italy, ethnobotanical studies are largely carried out in inland areas, since researchers usually presume that ethnobotanical knowledge is better preserved in these areas. Conversely, coastal areas are often neglected, as they are more densely urbanized. As a result, relatively few data on traditional uses of plants for fishery activities are available [27]. Fishing is also practiced in mountainous regions (lakes, rivers), but the techniques and plants used are different from marine fishery: for example the use of icthyotoxic plants for illegal fishing is frequently reported in fresh water basins, while it is rare in coastal areas [28].

All of the informants in our field research were men, since fishery and many fishery related activities are generally a male prerogative as in other parts of the world [6,14,15]. Some activities, such as the sewing of nets, are mainly the duty of women (Marina di Montalto, Aurunci [29]), Amalfi Coast, Aspra), and therefore the contribution of women in this activity was only related to us by these male informants. However, the sewing of nets is also a prerogative of elders (men and women) in various localities of Liguria and in Civitavecchia (Latium).

The wood of many tree species is used by shipwrights, and it is interesting to note that the number of species used for this purpose in the Western Mediterranean coastal regions of Italy is higher than in other parts of the world (e.g., [6,14,15,30]). The use of some species dates back to the period of the Roman Empire, such as the wood of *Quercus ilex* L. and *Quercus robur* L. or several coniferous species (especially pines) for building the hulls of ships [31]. The coastal habitat of some pines explains why in the past they were not only consecrated to Cybele, the divinity of fertility in Greco-Roman mythology (for the many seeds in their cones), but also to Poseidon, the god of the Sea [32].

Ligurian informants reported a method, named “*garibo*” in Savona, consisting of bending trees during growth by a weight fixed to the tip (e.g., *Pinus pinea* L., *Robinia pseudoacacia* L., etc.) to obtain boards for boat building (interviews; [33]). This practice has been reported in other Italian areas, but, to the best of our knowledge, not in other countries.

Species used for weaving ropes are generally the same in all the localities covered by our research. However, informants reported that certain native species (e.g., *Ampelodesmos mauritanicus*) have been replaced by exotic ones (e.g., *Cocos nucifera* L.) or by plastic materials (e.g., nylon). It is worth mentioning that *Cocos nucifera* is also used for weaving ropes in the Pacific [14]: its use in Italy may be both derived or a case of convergence.

With regards to *Ampelodesmos mauritanicus*, both informants and bibliographic sources [34-36] reported that this plant was also commercially important in the past, while today it is no longer used. This is probably due to the labor intensive process needed to make use of this plant. Leaves need to be dried, pounded, and macerated overnight, then pounded again and finally woven. As a result of this process the robust nature of the fibers are retained. Some species are chosen for different purposes, as for example in some Ligurian localities the fibers of *Cocos nucifera* are retained to attract fish such as the goldline (*Sarpa salpa*), probably because it eats algae that grow on these fibers. Another example given by some informants from Civitavecchia is that in the past, nets were made with cotton or hemp, and this fact was economically positive since worn out nets could then be sold to ragmen. Conversely, modern nylon is categorized as special waste and fishermen have to pay for its disposal.

Baskets, cages and fish traps using plant fibers are made from a relatively small number of plants, as in other regions in the world (e.g., [14]). This could be due to specific features the plants need to bear, or to the fact that these practices are steadily fading. Indeed, low-cost materials coming from Asian countries are now replacing traditional woven products [35,37]. On the other hand, fish traps, now made from plastic or metal materials, are more easily folded (Santa Marinella). Also, according to our informants, these modern products last longer (Santa Marinella, Civitavecchia), even if they are less effective (Amalfi Coast).

Despite a variability in the common names of plants (not reported here) in different dialects, the names of some artifacts are very similar. For example, the baskets made of chestnut fibers used for carrying fish are called “*cofone*” or “*cofuìn*” in Liguria and “*coffe*” in Campania. The cage (made of different plant species) used to keep fish alive is called “*maruffo*” in Ischia (Campania) [37] and “*maruffu*” in Sardinia [38]. The dye obtained by a decoction of *Pinus* bark was indicated as “*zappino*” or “*zappinu*” by informants from Liguria, and the same name occurred in Sardinia [38], Campania [27,37] and Sicily (interviews; [39]). This could be related to the ancient cultural connections among these regions showing that these uses are part of a shared cultural background. Conversely, in the Pacific it is retained that different plant names suggest the plants have been there for a long time, while having names of products and technologies in common suggest that these are relatively new and shared among practitioners in different islands. However, the historical and cultural backgrounds of countries of the Mediterranean are completely different from those of the Pacific.

The plants being used are essentially trees or bushes, growing mainly in the Mediterranean belt, likely because these plants are easier to stock up on. Some uses require a specific species while, for others, a species may be replaced by another. However, some species are specifically chosen: all the species of the temperate belt are used for shipbuilding, probably because the wood of the trees growing in this habitat has features that fit better for specific uses in shipbuilding [several species are selectively used for specific parts of the boat (e.g., *Pinus* species are only used for the hull) and a similar selectivity has been also reported in [14]]. This probably justifies the higher costs and labor involved in obtaining this resource. Natural habitats are the preferred place for gathering ethnobotanical species, especially Mediterranean woodland, maquis and garigues. Finally, it has been highlighted that changes in the gathering of certain species have modified the natural environment. For example, in Camogli (Liguria) the abandonment of the practices connected to the gathering of *Ampelodesmos mauritanicus,* adapted to a low frequency and medium intensity of fires, has led to an increase of this species, while in the Aurunci area (Latium), the same fact has caused an increase in fire hazard [35]. The higher number of fires is probably due to a reduction in maintenance of the resource and to a prevailing activity of shepherds who often start fires for obtaining new grass.

There is not an absolute and unilateral selection criterion for gathering plants: the correlations between use categories and the different considered variables seem to vary. In some cases the statistical analyses confirm and/or support ethnobotanical observations and ecological explanations, while in other cases, results are limited by data availability (which is a common problem in ethnobotanical research, especially for uses that are disappearing).

In some cases plants need to have certain specific physical features, a fact that has also been observed in other countries (e.g., [15,40]) like being woody for ship building and thus, these species are even gathered rather far from inland (e.g., in temperate woodland). In other cases, different plants can be used for the same purpose, and so there is no strong relation between a use and a certain variable (at least the ones that we analyzed). In other cases, the connection can be explained by the presence of certain compounds in the species like the use of several *Pinus* species (tannins) for “Dyeing of fish nets”.

The regions that share the higher number of uses are Liguria and Campania, but this may be explained by the fact that we were able to conduct a higher number of interviews in these regions. However, many uses are also shared between Campania and Sicily, and between Liguria and Sardinia. This may be the result of historical connections and reciprocal commercial activities in the past. Moreover, several regions are linked to a specific usage category, this is probably due to the fact that in some regions (e.g., Lucania and Calabria) available data were limited but also to other various factors: for example barrels are mainly made in Sicily for the practice of preserving fish under salt, which is much appreciated and consumed by local people.

**Figure 6:**
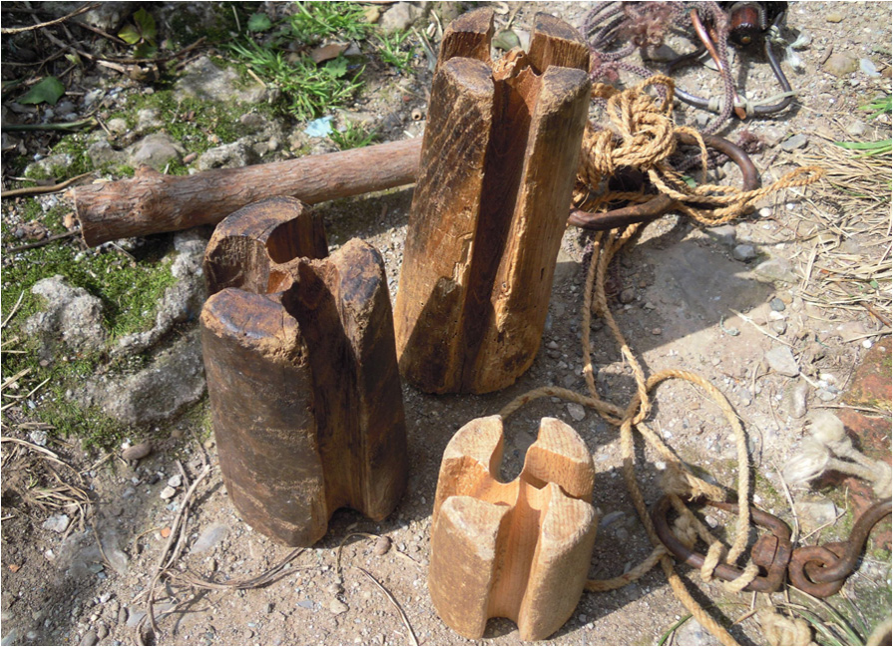
**Tools made of *Castanea sativa *wood (the two bigger ones).** These tools are used to twist the single threads for weaving ropes.

According to our informants, fishing activity is in reduction, while the local production of traditional artifacts is even rarer (Figure 6). Artisanal fishery is decreasing in the studied areas and according to our informants this reduction might depend on different causes: too many fishing vessels within the limit of 20 miles off the coast (Civitavecchia, Palermo), legal restrictions (Civitavecchia, Palermo), decline in fish stocks (Marina di Montalto, S. Marinella, Civitavecchia, Palermo), changes in fish species (Marina di Montalto, S. Marinella), climatic changes (specifically: different seasonality) (S. Marinella), dolphin predation (Aspra, Palermo), increases in cost (Civitavecchia, Palermo). Traditional handicrafts, practices and strategies are disappearing even faster, with the majority of plant uses having already been abandoned. Finally, the fact that these uses are disappearing makes it difficult to assess whether a specific use is unique to a certain area or if it has already disappeared in other areas.

## Conclusions

This paper, which provides a broad overview on plants used in artisanal fisheries in the Western Mediterranean coastal regions of Italy, underlines some correlations among functional features and habitats of plants, and discusses the sharing of their ethnobotanical uses. Plants for fishery activities are selected for different reasons, mainly for characteristics suitable for a specific use in surrounding habitats, as the chorological and structural analysis confirms. Ethnobotanical uses in fishery and its related knowledge are steadily disappearing in the majority of the investigated localities as is happening in other countries. Causes for this decline are different, as reported by our informants. Our contribution is to keep, at least, a few memories of this ethnobotanical knowledge.

## Competing interests

In the past five years have you received reimbursements, fees, funding, or salary from an organization that may in any way gain or lose financially from the publication of this manuscript, either now or in the future? No. Do you hold any stocks or shares in an organization that may in any way gain or lose financially from the publication of this manuscript, either now or in the future? No. Do you hold or are you currently applying for any patents relating to the content of the manuscript? No. Have you received reimbursements, fees, funding, or salary from an organization that holds or has applied for patents relating to the content of the manuscript? No. Do you have any other financial competing interests? No. Are there any non-financial competing interests (political, personal, religious, ideological, academic, intellectual, commercial or any other) to declare in relation to this manuscript? No.

## Authors’ contributions

VS collected data in central-southern Italy, participated in the analysis of data and drafted the manuscript. LA collected data in northern Italy and analyzed the data. GC participated in the design of the study and supervised the research in central-Southern Italy. FR performed the statistical analysis of data and participated in the drafting of the manuscript. LC collected data and supervised research in northern Italy and participated in the drafting of the manuscript. All authors have read and approved the final manuscript.

## Supplementary Material

Additional file 1 **Plant uses with relative details (plant part, source of data, localities, status of the use) [**[41-53]**].** Click here for file
