# Supplementary material for: Plants used in artisanal fisheries on the Western Mediterranean coasts of Italy
Source: J Ethnobiol Ethnomed. 2013 Jan 28;9:9. doi: 10.1186/1746-4269-9-9 (PMC3570400; doi:10.1186/1746-4269-9-9)
Supplement: Additional file 1 — Plant uses with relative details (plant part, source of data, localities, status of the use) [[41-53]]. [file 1746-4269-9-9-S1.pdf]

Species	Plant part	Use	Status	Region	Locality	N° of cit.	Source	Description of the use
Acacia karroo Hayne	Thorns	Hooks	N/A	Sardinia	Cabras	N/A	[38]	Hooks (for fishing pole)
Agave americana L.	Stalk	Ship building	N/A	Sicily	Pantelleria	N/A	[41]	Light beams and masts (for small ships)
	Leaves	Ropes	N/A	Sicily	Riserva dello Zingaro	N/A	[42]	Ropes for tuna nets
Alnus glutinosa (L.) Gaertn.	Bark	Dyeing of fish nets	O	Tuscany	Massaciuccoli	N/A	[43]	The decoction of the bark was used to dye and strengthen fish nets
	Wood	Ship building	N/A	Liguria	Savona	N/A	[33]	Upperworks of ships
Ampelodesmos mauritanicus (Poiret) T. Durand et Schinz	Leaves	Ropes, fish nets	O	Liguria	Genova, Arenzano	N/A	[44;45]	Ropes (“*cavi d’erba*” or “*libani*”) for a typical net ("*tartanone*" or “*gangano*”)
	Leaves	Ropes, fish nets	O	Liguria	Camogli	3	Interviews	Ropes and fish nets (tuna nets)
	Leaves	Ropes, fish nets	O	Campania	Praiano, Furore, Conca dei Marini	4	Interviews/ [27;46]	Ropes (mussel farming) and tuna nets
	Leaves	Ropes, fish traps	O	Latium	Formia	N/A	[29]	Ropes (for fish nets and lobster traps)
	Leaves	Basketry	C(O)	Latium	Formia	N/A	[29,35]	Trays and baskets for carrying fish
	Leaves	Fish nets, ropes	C	Liguria	Camogli, Recco	4	Interviews	Typical fish net ("*trattanùn*" or "*trattanone*") for traditional trawling fishing. Ropes (for tying boats)
	Leaves	Ropes	O	Lucania	Maratea	N/A	[36]	Ropes (for tying boats)
	Leaves	Ropes	O	Lucania	Maratea	N/A	[36]	Ropes (mussel farming)
	Leaves	Ropes	N/A	Sicily	Riserva dello Zingaro	N/A	[42]	Ropes (for tuna nets)
	Stems	Ropes	O	Sicily	Aspra	1	Interviews	Various types of ropes
	Leaves	Ropes/mussel farming	N/A	Liguria	La Spezia	N/A	[44]	Ropes (mussel farming)
Arbutus unedo L.	Wood	Ship	N/A	Liguria	Camogli, Recco	4	Interviews	Oarlocks

		building						
	Wood	Tools	N/A	Liguria	Camogli	2	Interviews	Needles ("*aguggia*") for repairing nets
Arundo donax L.	Stems	Fish traps	O	Campania	Ischia	N/A	[37]	Fish traps
	Stems	Fish traps	O	Sardinia	Various locations	N/A	[38]	Fish traps (with other plant species)
	Stems	Basketry	O	Campania	Ischia	N/A	[37]	Baskets ("*maruffo*") for keeping fish alive
	Stems	Tools	O	Campania	Ischia	N/A	[37]	Needles ("*cucelle*") for weaving and repairing nets
	Stems	Fish nets	O	Campania	Ischia	N/A	[37]	Sustaining structure of small fish nets
	Stems	Dams of ponds	N/A	Sardinia	Various locations	N/A	[38]	Dams of ponds (aquaculture)
	Stems	Ship building	O	Sardinia	Cabras, Santa Giusta	N/A	[38]	Parts of small ships for brackish water basins
Arundo plinii Turra	Stems	Fish traps	O	Campania	Ischia	N/A	[37]	Fish traps
	Stems	Basketry	O	Campania	Ischia	N/A	[37]	Baskets ("*maruffo*") for keeping fish alive
Brassica oleracea L.	Leaves	Baits	N/A	Liguria	Camogli	1	Interviews	Bait in fish traps
Cannabis sativa L. .	Fibers	Fish nets	O	Sicily	Aspra	1	Interviews	Fish net ("*conzo*") part of the "*palancaro*", for fishing sea urchins and shrimps
	Fibers	Fish nets	C(O)	Sardinia	Various location	N/A	[38]	Fish nets (e.g., tuna nets and the *"palamiti*")
	Stems	Fish nets	C	Liguria	Camogli, Pieve Ligure	2	Interviews	Fish nets, especially the death chamber ("*u cagnu*") of tuna nets
	Fibers	Fish nets	O	Latium	Civitavecchia	2	Interviews	Fish nets. In the past, fish nets were made of cotton or hemp
	Stems	Fish nets, ropes	O	Liguria	Savona	3	Interviews	Ropes or fish nets (e.g., the "*sciàbiche*")
	Fibers	Ropes	C	Sicily	Aspra	1	Interviews	Ropes
	Fibers	Ropes	O	Sardinia	Various location	N/A	[38]	Ropes
	Stems	Ropes	C	Liguria	Imperia Oneglia	1	Interviews	Ropes
	Stems	Ropes	N/A	Liguria	Camogli	1	Interviews	Ropes (e.g., the "*spiòrsi*": the ropes used to secure multiple boats)
	Stems	Caulking	O	Liguria	Camogli, Pieve Ligure, Recco	3	Interviews	Caulking. Fibers are introduced in the cracks of the hull
	Stems	Caulking	O	Liguria	Imperia Oneglia	1	Interviews	Caulking. Fibers are introduced in the cracks of the hull

	Stems	Caulking	O	Liguria	Savona	3	Interviews	Calking. Fibers and pitch (the "*stuppa*")
Castanea sativa Miller	Wood	Basketry	N/A	Liguria	Genova	N/A	[44]	Baskets ("*panêa*") for carrying anchovies
	Wood	Basketry	C	Liguria	Camogli, Pieve Ligure	2	Interviews	Baskets ("*cofone*" or "*cofuìn*") for carrying fish
	Wood	Grill	N/A	Liguria	Camogli, Recco	2	Interviews	Grill used to dry fish nets
	Wood	Tools	O	Liguria	Camogli	1	Interviews	Tool ("*pigna*") for weaving ropes
	Wood	Fish nets, Fish traps	O	Liguria	Noli	1	Interviews	Lobster traps
	Wood	Ship building	N/A	Liguria	Savona	N/A	[33]	Ship masts
	Wood	Ship building	O	Campania	Amalfi	2	Interviews/ [27;46]	Upperworks of ships
	Wood	Barrels	C	Campania	Cetara	3	Interviews	Barrels for preserving sardines under salt
	Wood	Barrels	C	Sicily	Palermo, Aspra	2	Interviews	Barrels ("*varrili*") for preserving fish under salt
	Wood, Bark	Basketry	C(O)	Campania	Amalfi, Minori	2	Interviews/ [27;46]	Baskets ('*coffe*') for cod fishing or for carrying fish
	Bark	Dyeing of fish nets	N/A	Liguria	Savona	1	Interviews	The decoction of the bark (with other species) is used to dye fish nets
	Root suckers	Barrels	O	Liguria	Pieve Ligure	1	Interviews	Structure of barrels for anchovies
	Trunk	Mussel farming	O	Campania	Tramonti	1	Interviews	Stakes for mussel farming
	Trunk	Mussel farming	O	Sardinia	Olbia	N/A	[38]	Stakes for mussel farming
Ceratonia siliqua L.	Wood	Ship building	C(O)	Campania	Amalfi, Cetara, Maiori	6	Interviews/ [27;46]	Transverse frames of ships
Chamaerops humilis L.	Fibers	Fish nets	N/A	Sardinia	North of Sardinia	N/A	[38]	Fish nets
	Leaves	Shrouds, ropes	C(O)	Sardinia	Various locations	N/A	[38]	Ropes and shrouds
	Leaves	Fishing	C	Sicily	Aspra	1	Interviews	Leaves used to make shadows on the water to attract fish
Citrus aurantium L.	Wood	Ramps	O	Campania	Amalfi	1	Interviews/ [46]	Ramps ("*falanghe*") for pulling small ships out of the water
Citrus limon (L.) Burm. f.	Wood	Ramps	C(O)	Campania	Amalfi, Minori	2	Interviews/ [27;46]	Ramps ("*falanghe*") for pulling small ships out of the water

Cocos nucifera L.	Husk of seeds (Coir)	Fish nets	C	Liguria	Camogli	1	Interviews	Fish nets (tuna nets)
	Husk of seeds	Fish nets	N/A	Liguria	Camogli	N/A	[44]	Fish nets (tuna nets)
	Husk of seeds	Fish nets	N/A	Liguria	Noli	2	Interviews	Special fish net ("*tartanùn*") for fishing species of sandy bottoms
	Husk of seeds	Ropes	C	Liguria	Camogli	3	Interviews	Ropes (for tuna nets)
	Husk of seeds	Ropes	C	Liguria	Imperia Oneglia	1	Interviews	Thick ropes (as bumpers for ships)
	Husk of seeds	Ropes	O	Liguria	Savona	1	Interviews	Ropes for gathering coral. They were tied to a typical wooden cross ("*ingegno*")
	Husk of seeds	Ropes	O	Sardinia	Various locations	N/A	[38]	Ropes for tuna nets
	Husk of seeds	Ropes	O	Sicily	Aspra	1	Interviews	Ropes
Cormus domestica (L.) Spach	Wood	Ramps	C	Campania	Amalfi	1	Interviews/ [27;46]	Ramps ("*falanghe*") for pulling small ships out of the water
	Wood	Ship building	C(O)	Campania	Amalfi	1	Interviews/ [27;46]	Keel of ships
Daphne gnidium L.	Plant	Illegal fishing	O	Tuscany	Maremma	N/A	[47]	Illegal fishing
Erica arborea L.	Branches	Fish traps	O	Campania	Ischia	N/A	[37]	Fish traps
	Branches	Basketry	O	Campania	Ischia	N/A	[37]	Baskets ("*maruffo*") for keeping fish alive
Euphorbia characias L.	Aerial parts	Fishing	N/A	Liguria	Noli	1	Interviews	The plant is crushed and then submerged close to caves in order to drive out fish
Euphorbia dendroides L.	Fresh branches	Illegal fishing	N/A	Sicily	Ustica	N/A	[39]	Illegal fishing
	Latex	Illegal fishing	N/A	Sicily	Pantelleria	N/A	[41]	Illegal fishing. People believe that the plant blinds fish which can then be scooped up with nets
Euphorbia helioscopia L.	Fresh branche	Illegal fishing	N/A	Sicily	Ustica	N/A	[39]	Illegal fishing

	s							
Fagus sylvatica L.	Wood	Barrels	O	Liguria	Savona	1	Interviews	Barrels for preserving anchovies
	Wood	Barrels	O	Liguria	Pieve Ligure	1	Interviews	Barrels for preserving anchovies
	Wood	Ship building	C	Liguria	Camogli, Pieve Ligure, Recco	4	Interviews	Oars
	Wood	Ship building	N/A	Liguria	Savona	1	Interviews	Upperworks of ships
	Wood	Ship building	N/A	Liguria	Imperia Oneglia	1	Interviews	Oars (because the wood is without nodes)
	Wood	Ship building	O	Campania	Amalfi	1	Interviews/ [27;46]	Oars
	Wood	Tools	O	Liguria	Camogli	1	Interviews	A tool ("*pigna*") for weaving ropes
Fraxinus ornus L.	Branches	Fish traps	O	Liguria	Noli	1	Interviews	Lobster traps
	Wood	Barrels	O	Sicily	Aspra	1	Interviews	Barrels ("*varrili*") for preserving sardines under salt
	Wood	Ship building	N/A	Liguria	Genova	N/A	[44]	The keel and posts, but also curved parts of the ships (transverse frames, borders)
	Wood	Ship building	C(O)	Liguria	Bogliasco, Camogli, Pieve Ligure, Recco	9	Interviews	The keel and posts, but also curved parts of the ships (transverse frames, borders)
	Wood	Ship building	C	Liguria	Pieve Ligure	2	Interviews	Oars and oarlocks
	Wood	Ship building	N/A	Liguria	Imperia Oneglia	1	Interviews	Oars and oarlocks, small parts of ships
	Wood	Ship building	N/A	Liguria	Imperia Oneglia	2	Interviews	Upperworks and transverse frames. Wood has to be boiled
	Wood	Ship building	N/A	Liguria	Savona	N/A	[33]	Upperworks and curved parts of ships
	Wood	Ship building	C	Liguria	Savona	1	Interviews	Oarlocks
	Wood	Ship building	O	Liguria	Savona	3	Interviews	Timber for ship building. Wood was curved using hot water
	Wood	Tools	N/A	Liguria	Camogli	2	Interviews	Needles for weaving and repairing nets
Fraxinus ornus L. subsp. *ornus*	Wood	Ship building	O	Campania	Praiano	1	Interviews/ [27;46]	Ship masts
	Wood	Ship	C(O	Campania	Amalfi, Cetara,	4	Interviews/ [27;46]	Oarlocks and upperworks

		building	)		Minori, Praiano			
Gossypium sp. pl.	Fibers	Fishing	N/A	Liguria	Genova	N/A	[44]	Fish nets for the “*lampara*”
	Fibers	Fishing	O	Campania	Amalfi	1	Interviews	Fibers were used to tie anchovies (baits) for fishing squid
	Fibers	Fishing	O	Liguria	Camogli	1	Interviews	Fish nets for the “*lampara*”
	Fibers	Fish traps	O	Sardinia	Cabras	N/A	[38]	Parts of fish traps
	Fibers	Fish nets	O	Sardinia	Cabras, La Maddalena	N/A	[38]	Nets for tuna fish and gathering coral
	Fibers	Fish nets	O	Latium	Civitavecchia	1	Interviews	Fish nets
	Fibers	Fish nets	O	Latium	Marina di Montalto	1	Interviews	Fish nets
	Fibers	Fish nets	O	Latium	Santa Marinella	1	Interviews	Fish nets
	Fibers	Fish nets	O	Sicily	Palermo	1	Interviews	Fish nets of different dimensions
	Fibers	Fish nets	O	Campania	Amalfi, Cetara, Praiano	4	Interviews	Fish nets (e.g., the “*ciancola*” for anchovies)
	Fibers	Fish nets	N/A	Liguria	Camogli	1	Interviews	Fish nets (e.g., the "*manàta*")
	Fibers	Fish nets	O	Liguria	Savona	1	Interviews	Nets of the “*lampara*”
	Fibers	Fish nets	O	Liguria	Noli	1	Interviews	Fish nets (nets for trawling or gathering coral)
	Fibers	Fish nets, fish traps	O	Liguria	Noli	1	Interviews	Fish traps (for lobsters)
	Fibers	Fish traps	O	Latium	Civitavecchia	1	Interviews	Some parts of fish traps
	Fibers	Caulking	O	Liguria	Savona	1	Interviews	Caulking ("*canapa impacciata"*)
	Fibers	Caulking	O	Campania	Amalfi	1	Interviews	Caulking
	Fibers	Caulking	C	Liguria	Pieve Ligure	1	Interviews	Caulking
	Fibers	Caulking	N/A	Liguria	Imperia Oneglia	1	Interviews	Caulking of small ships
Helichrysum italicum (Roth) G. Don	Plant	Baits	O	Liguria	Riomaggiore	N/A	[48]	The plant was used to smoke octopi (baits)
	Plant	Baits	O	Sardinia	La Maddalena	N/A	[38]	The plant was used to smoke octopi (baits)
Juglans regia L.	Wood	Ship building	C	Liguria	Recco	1	Interviews	Keel and posts of ships
	Wood	Ship building	C	Liguria	Savona	N/A	[33]	Decorations on ships (e.g., the figurehead)
	Wood	Ship building	N/A	Liguria	Savona	1	Interviews	Oars
Juncus sp.	Stems	Fish traps	O	Liguria	Camogli	1	Interviews	Fish traps (for lobsters)
	Stems	Fish traps	O	Latium	Civitavecchia	1	Interviews	Fish traps

	Stems	Fish traps	O	Campania	Conca dei Marini	1	Interviews	Fish traps of different shapes
	Stems	Fish traps	C	Sicily	Palermo	1	Interviews	Fish traps (crabs used as bait)
Juncus sp. pl.	Stems	Fish traps	O	Campania	Ischia	N/A	[37]	Fish traps
	Stems	Basketry, fish traps	C(O)	Sardinia	Various locations	N/A	[38]	Fish traps and cages to keep fish alive
	Stems	Basketry	O	Campania	Ischia	N/A	[37]	Baskets (“*maruffo*”) for keeping fish alive
	Stems	Ropes	C(O)	Sardinia	Various locations	N/A	[38]	Ropes
Juncus acutus L.	Stems	Fish traps	N/A	Sardinia	Various locations	N/A	[38]	Fish traps
	Stems	Ship building	N/A	Sardinia	Cabras, Santa Giusta	N/A	[38]	Parts for small ships that are used for fishing in brackish water basins
Juncus maritimus Lam.	Stems	Fish traps	C	Sardinia	Alghero and other locations	N/A	[38]	Fish traps
Juniperus sp. pl.	Wood	Ship building	O	Sardinia	Various locations	N/A	[38]	Timber for ship building
	Branches	Fishing	O	Sardinia	Gallura, Iglesias, Sulcis	N/A	[38]	Torches (for attracting fish at night)
Laburnum anagyroides Medik	Wood	Ship building	C	Liguria	Pieve Ligure	1	Interviews	Transverse frames of small ships (the trunk is irregular)
	Wood	Ship building	N/A	Liguria	Savona	N/A	[33]	Timber for ship building
Larix decidua Mill.	Wood	Ship building	C	Liguria	Imperia Oneglia	1	Interviews	Hull (small ships)
	Wood	Ship building	N/A	Liguria	Savona	N/A	[33]	Hull
	Wood	Ship building	N/A	Liguria	Imperia Oneglia	1	Interviews	Upperworks of ships
Laurus nobilis L.	Branches	Fishing	O	Campania	Pozzuoli	1	Interviews	Branches were used to make shadows on the water to attract fish
Linum usitatissimum L.	Seeds/Oil	Waterproofing	N/A	Liguria	Recco	1	Interviews	Seed oil is used as a waterproofing agent (for masts)
	Seeds/Oil	Coloring	C	Campania	Amalfi	1	Interviews/ [46]	Seed oil is used for brightening colors (of the wood or of paint
	Seeds/Oil	Coloring	C	Sicily	Aspra	1	Interviews	Seed oil is used to paint the hull

Lygeum spartum L.	Stem	Ropes/fish nets	O	Sardinia	Iglesiente, Asinara	N/A	[38]	Ropes and tuna nets
Mespilus germanica L.	Wood	Ship building	C	Sicily	Aspra	1	Interviews	Upperworks, especially the trim of the hull
Morus sp.	Wood	Ship building	O	Calabria	Scilla and Bagnara areas	N/A	[49]	Timber for ship building
	Wood	Ship building	O	Liguria	Savona	N/A	[33]	Ship masts
Morus alba L.	Wood	Ship building	C(O)	Campania	Amalfi, Vietri	2	Interviews/ [27;46]	Transverse frames and upperworks of ships
	Wood	Ship building	C	Sicily	Aspra	1	Interviews	Transverse frames of small ships
Myrtus communis L.	Branches	Fish traps	O	Latium	Formia	N/A	[29;35]	Fish traps
	Branches	Fish traps	C	Sardinia	Various locations	N/A	[38]	Fish traps of different sizes and shapes
	Branches	Basketry	C	Sardinia	Various locations	N/A	[38]	Baskets (“*marruffu*”) or cages for keeping fish alive
	Plant	Baits	N/A	Campania	Ischia	N/A	[50]	The plant is used to smoke octopi (baits for catching moray eels)
	Wood	Tools	C	Sardinia	Santa Teresa di Gallura	N/A	[38]	Spool for cotton strings
Myrtus communis L. subsp. *communis*	Branches	Fish traps	O	Campania	Amalfi, Conca dei Marini	3	Interviews/ [27;46]	Fish traps (different shapes)
Olea europaea L.	Branches	Basketry	O	Sardinia	Cabras	N/A	[38]	Baskets (“*su kadinu*”) for carrying fish
	Branches	Fish traps	O	Campania	Conca dei Marini, Praiano	3	Interviews/ [27;46]	Fish traps of different shapes
	Wood	Ship building	C	Liguria	Pieve Ligure	1	Interviews	Transverse frames of ships
	Wood	Tools	C	Latium	Marina di Montalto	1	Interviews	Needles (“*cucelle*”) for weaving and repairing nets
	Wood	Ramps	C	Campania	Praiano	1	Interviews/ [27;46]	Ramps (“*falanghe*”) for pulling small ships out of the water
	Wood	Ship building	O	Sardinia	Stintino, Asinara	N/A	[38]	Timber for ship building

	Wood	Fish traps	O	Sardinia	Various localities	N/A	[38]	Fish traps of different sizes and shapes
	Oil	Fishing	O	Sicily	Aspra	1	Interviews	The oil was used to light a candle ("*a cannila*"), which was part of a night fishing tool
	Oil	Fishing	O	Sardinia	Tresnuraghes	N/A	[38]	Drops of oil were poured on the water for keeping the surface clear while fishing octopi
Opuntia ficus-indica (L.) Miller	Cladodes	Making ships going faster	O	Campania	Conca dei Marini	1	Interviews/ [46]	Cladodes were rubbed on the hull of ships to make them go faster
Phillyrea angustifolia L.	Branches	Tools	O	Sardinia	La Maddalena, Caprera	N/A	[38]	Needles ("*cucelle*") for weaving and repairing nets
Phormium tenax J.R. et G. Forster	Fibers	Fish nets, ropes	O	Sardinia	Villa d'Orri	N/A	[38]	Ropes and fish nets
Phragmites australis (Cav.) Trin.	Stems	Dams of ponds	O	Sardinia	Sinis area	N/A	[38]	Dams of ponds (aquaculture in brackish water)
Picea abies (L.) H. Karst.	Wood	Ship models	O	Liguria	Recco	1	Interviews	Ship models
	Wood	Ship building	C	Liguria	Pieve Ligure, Recco	2	Interviews	Oars
	Wood	Ship building	N/A	Liguria	Savona	N/A	[33]	Hull
Pinus sp.	Bark	Dyeing of fish nets	O	Liguria	Savona	2	Interviews	The decoction of the bark was used to dye and strengthen fish nets
	Bark	Dyeing of fish nets	O	Sardinia	La Maddalena, Santa Teresa di Gallura	N/A	[38]	The decoction of pine ("*zappino*") bark was used to dye and strengthen fish nets
	Wood	Ship building	C	Liguria	Imperia Oneglia	2	Interviews	Hull (small ships)
	Wood	Ship building	N/A	Liguria	Savona	N/A	[33]	Hull
	Wood	Ship building	O	Liguria	Savona	2	Interviews	Timber for ship building
	Wood	Ship building	C	Liguria	Recco	1	Interviews	Oars

	Wood	Ship building	C	Latium	Marina di Montalto	1	Interviews	Hull
	Wood	Ship building	O	Calabria	Scilla and Bagnara areas	N/A	[49]	Timber for ship building
Pinus sp. pl.	Bark	Dyeing of fish nets	O	Sicily	Aspra	1	Interviews	The decoction of pine ("*zappino*") bark was used to dye fish nets
	Bark	Dyeing of fish nets	O	Campania	Pozzuoli	1	Interviews	The decoction of the bark was used to dye fish nets
	Bark	Dyeing of fish nets	O	Latium	Santa Marinella	1	Interviews	The decoction of the bark was used to dye fish nets
Pinus halepensis Mill.	Bark	Dyeing of fish nets	O	Campania	Ischia	N/A	[37]	The decoction ("*zappino*") of the bark was used to dye fish nets
	Bark	Dyeing of fish nets	O	Sicily	Alicudi, Lipari, Panarea, Stromboli, Vulcano	N/A	[51]	The decoction of the bark was used to dye fish nets
	Wood	Ship building	O	Sardinia	Carloforte and Sant'Antioco Islands	N/A	[38]	Timber for ship building
	Wood	Ship building	C	Sicily	Pantelleria	N/A	[41]	Timber for ship building
Pinus nigra J.F. Arnold s.l.	Wood	Ship building	O	Sardinia	Various locations	N/A	[38]	Timber for ship building
Pinus pinaster Aiton s.l.	Wood	Ship building	O	Liguria	Camogli, Pieve Ligure	4	Interviews	Timber for ship building
Pinus pinea L.	Bark	Dyeing of fish nets	O	Liguria	Bogliasco, Camogli, Recco	9	Interviews	The decoction of the bark was used to dye and strengthen fish nets
	Bark	Dyeing of fish nets	O	Campania	Praiano	1	Interviews/ [27;46]	The decoction of the bark was used to dye fish nets
	Bark	Dyeing of fish nets	O	Campania	Ischia	N/A	[37]	The decoction ("*zappino*") of the bark was used to dye fish nets
	Bark	Dyeing of fish nets	C	Liguria	Noli	1	Interviews	The decoction of the bark was used to dye and strengthen fish nets
	Wood	Ship building	C	Liguria	Savona	3	Interviews	Hull. Wood is curved using steam or trees are forced to grow curved
	Wood	Ship building	C(O)	Liguria	Bogliasco, Camogli, Recco	5	Interviews	Hull. The seasoned wood is resinous and easy to bend

	Wood	Ship building	C(O)	Campania	Amalfi, Vietri	5	Interviews/ [27;46]	Hull
Pistacia lentiscus L.	Branches	Fish traps	O	Campania	Conca dei Marini	1	Interviews/ [27;46]	Fish traps
	Branches	Fish traps	O	Campania	Ischia	N/A	[37]	Fish traps
	Branches	Basketry	O	Campania	Ischia	N/A	[37]	Baskets ("*maruffo*") for keeping fish alive
	Branches	Basketry	O	Sardinia	Various locations	N/A	[38]	Fish traps, baskets ("*maruffo*") and cages for lobsters
	Green branches	Dyeing of fish nets	C	Sicily	Pantelleria	N/A	[52]	The decoction of green branches is used to dye fish nets
	Green branches	Dyeing of fish nets	O	Sicily	Mazara del Vallo	N/A	[53]	Green branches of the plant were macerated in water (used to dye fish nets and other fishing tools)
	Leafy branches	Illegal fishing	O	Tuscany	Maremma	N/A	[47]	Illegal fishing
Prunus avium (L.) L.	Wood	Ship building	N/A	Liguria	Camogli, Recco	2	Interviews	Oars and oarlocks
Punica granatum L.	Branches	Fish traps	O	Sardinia	Cabras	N/A	[38]	Fish traps
Pyrus communis L.	Wood	Pulleys	N/A	Liguria	Noli	1	Interviews	Pulleys ("*paranchi*") for pulling ships out of the water
Quercus sp.	Wood	Ship building	N/A	Liguria	Imperia Oneglia	1	Interviews	Transverse frames of ships
	Wood	Ship building	N/A	Liguria	Savona	N/A	[33]	Transverse frames of ships
	Wood	Ship building	N/A	Liguria	Imperia Oneglia	1	Interviews	Hull (inside and outside)
	Wood	Ship building	N/A	Liguria	Camogli	1	Interviews	Keel of ships
	Wood	Ship building	O	Latium	Civitavecchia		Interviews	Timber for ship building
	Wood	Ship building	C	Latium	Marina di Montalto	1	Interviews	Transverse frames of ships

	Wood	Ship building	O	Calabria	Scilla and Bagnara areas	N/A	[49]	Timber for ship building
Quercus ilex L.	Trunk	Mussel farming	O	Sardinia	Olbia	N/A	[38]	Stakes for mussel farming
	Wood	Ship building	O	Liguria	Camogli, Recco	2	Interviews	Timber for ship building
	Wood	Ship building	C	Liguria	Recco	2	Interviews	Keel of ships
	Wood	Ship building	N/A	Liguria	Genova	1	Interviews	Transverse frames of ships
	Wood	Ramps	O	Campania	Praiano	1	Interviews/ [27;46]	Ramps ("*falanghe*") for pulling small ships out of the water
	Wood	Ramps	C	Sicily	Pantelleria	N/A	[41]	Ramps ("*falanghe*") for pulling small ships out of the water
	Wood	Ship building	C(O)	Campania	Amalfi, Vietri	3	Interviews/ [27;46]	Transverse frames of ships, the keel and curved parts of upperworks
Quercus pubescens Willd. s.l.	Wood	Barrels	O	Sicily	Aspra		Interviews	Barrels ("*varrili*") for preserving sardines under salt
Quercus pubescens Willd. subsp. *pubescens*	Wood	Ship building	C(O)	Campania	Amalfi, Cetara, Praiano, Vietri	4	Interviews/ [27;46]	Transverse frames, upperworks and the keel
	Wood	Ramps	O	Campania	Praiano	1	Interviews/ [27;46]	Ramps ("*falanghe*") for pulling small ships out of the water
Quercus robur L. s.l.	Wood	Ship building	N/A	Liguria	Imperia Oneglia	1	Interviews	Hull (inside and outside) of big boats
	Wood	Ship building	N/A	Liguria	Savona	3	Interviews	Transverse frames of ships
	Wood	Ship building	N/A	Liguria	Savona	2	Interviews	Oarlocks
	Wood	Ship building	N/A	Liguria	Savona	1	Interviews	Hull
	Wood	Ship building	N/A	Liguria	Savona	1	Interviews	Oars
	Wood	Ship building	O	Liguria	Camogli	1	Interviews	Straight parts of ships (e.g., "*a cuvèrta de rùvia*")
	Wood	Ship building	C	Liguria	Pieve Ligure	2	Interviews	Keel of ships

	Wood	Ship building	C	Liguria	Pieve Ligure	2	Interviews	Transverse frames of ships
	Wood	Ship building	C	Liguria	Pieve Ligure	1	Interviews	Oarlocks
	Wood	Barrels	O	Sicily	Aspra		Interviews	Barrels (“*varrili*”) for preserving sardines under salt
Quercus suber L.	Bark	Basketry	C	Sardinia	Alghero	N/A	[38]	Trim of baskets (for keeping hooks)
	Bark	Basketry	C	Liguria	Pieve Ligure	1	Interviews	Trim of baskets (for keeping hooks)
	Bark	Floats	N/A	Liguria	Savona	1	Interviews	Floats for fishing pole
	Bark	Floats	O	Liguria	Camogli	2	Interviews	Floating signals for tuna nets
	Bark	Floats	O	Latium	Santa Marinella	1	Interviews	Floats. In the past, floats were carved by hand from the bark
	Bark	Floats	O	Campania	Ischia	N/A	[37]	Floats (“*natelli*”) for fish nets
	Bark	Floats	O	Sicily	Aspra		Interviews	Floats (“*sumo*”) for fish nets
	Bark	Floats	O	Sicily	Palermo	1	Interviews	Floats
	Bark	Floats	C	Sardinia	Alghero	N/A	[38]	Floats for fish nets and fish traps
Robinia pseudoacacia L.	Wood	Ship building	N/A	Liguria	Genova	N/A	[44]	Keel but also curved parts of ships (transverse frames, borders)
	Wood	Ship building	C	Liguria	Camogli, Pieve Ligure, Recco	3	Interviews	Hull and transverse frames (*“stamanèe”*) of ships. Wood is boiled and then curved using a special tool ("*cegùn*")
	Wood	Ship building	N/A	Liguria	Camogli, Recco	2	Interviews	Oars
	Wood	Ship building	C	Liguria	Pieve Ligure	1	Interviews	Oarlocks
	Wood	Ship building	C	Liguria	Recco	1	Interviews	Keel and stempost of ships
	Wood	Ship building	N/A	Liguria	Imperia Oneglia	1	Interviews	Hull (inside and outside) of big ships
Salix sp.	Branches	Barrels	O	Sicily	Aspra	1	Interviews	Structure of barrels (“*varrili*”) for preserving sardines under salt
	Branches	Fish traps	N/A	Liguria	Savona	1	Interviews	Fish traps (for lobsters)
	Branches	Fish traps	N/A	Liguria	Camogli	1	Interviews	Fish traps (for lobsters)
	Branches	Fish traps	N/A	Liguria	Imperia Oneglia	1	Interviews	Fish traps

Salix alba L.	Branches	Basketry	N/A	Liguria	Noli	2	Interviews	Baskets (for carrying the "*palamiti*")
	Branches	Fish traps	O	Campania	Amalfi	1	Interviews/ [27;46]	Fish traps
Schoenoplectus lacustris (L.) Palla	Stems	Basketry	O	Sardinia	Oristano area	N/A	[38]	Special basket used for fishing
	Stems	Ship building	C(O)	Sardinia	Cabras, Santa Giusta	N/A	[38]	Small traditional ships (used in brackish water basins)
Tamarix africana Poir.	Branches	Tools	O	Sardinia	Cabras	N/A	[38]	Fishing tool for catching shrimps and small fish species
Tamarix gallica L.	Branches	Basketry	O	Campania	Ischia	N/A	[37]	Baskets ("*maruffo*") for keeping fish alive
	Branches	Fish traps	O	Campania	Ischia	N/A	[37]	Fish traps
	Branches	Tools	O	Sardinia	Cabras	N/A	[38]	Fishing tool for catching shrimps and small fish species
Ulmus minor Miller s.l.	Wood	Ship building	C	Liguria	Pieve Ligure	1	Interviews	Transverse frames of ships
	Wood	Ship building	C	Liguria	Recco	1	Interviews	Keel and posts of ships
	Wood	Ship building	N/A	Liguria	Imperia Oneglia	1	Interviews	Hull and upperworks of ships
	Wood	Ship building	N/A	Liguria	Savona	N/A	[33]	Upperworks of ships
	Wood	Ship building	C	Campania	Vietri	1	Interviews/ [46]	Transverse frames of ships
Verbascum thapsus L.	Plant	Illegal fishing	O	Tuscany	Maremma	N/A	[47]	Illegal fishing

Notes: N/A: Not Available; O: Obsolete use; C: Currently in use. N° of cit: number of citations=number of times a plant has been mentioned in the same locality.
